# Spinal cord gray matter segmentation using deep dilated convolutions

**DOI:** 10.1038/s41598-018-24304-3

**Published:** 2018-04-13

**Authors:** Christian S. Perone, Evan Calabrese, Julien Cohen-Adad

**Affiliations:** 1 NeuroPoly Lab, Institute of Biomedical Engineering, Polytechnique Montreal, Montreal, QC H3T 1J4 Canada; 2 Duke University Medical Center, Department of Radiology, Center for In Vivo Microscopy, Durham, NC 27710 USA; 3 University of California San Francisco, Department of Radiology & Biomedical Imaging, San Francisco, CA 94143 USA; 4 Functional Neuroimaging Unit, CRIUGM, Université de Montréal, Montreal, QC H3C 3J7 Canada

## Abstract

Gray matter (GM) tissue changes have been associated with a wide range of neurological disorders and were recently found relevant as a biomarker for disability in amyotrophic lateral sclerosis. The ability to automatically segment the GM is, therefore, an important task for modern studies of the spinal cord. In this work, we devise a modern, simple and end-to-end fully-automated human spinal cord gray matter segmentation method using Deep Learning, that works both on *in vivo* and *ex vivo* MRI acquisitions. We evaluate our method against six independently developed methods on a GM segmentation challenge. We report state-of-the-art results in 8 out of 10 evaluation metrics as well as major network parameter reduction when compared to the traditional medical imaging architectures such as U-Nets.

## Introduction

Gray matter (GM) and white matter (WM) tissue changes in the spinal cord (SC) have been linked to a large spectrum of neurological disorders^1^. For example, using magnetic resonance imaging (MRI), the involvement of the spinal cord gray matter (SCGM) area in multiple sclerosis (MS) was found to be the strongest correlate of disability in multivariate models including brain GM and WM volumes, FLAIR lesion load, T1-lesion load, SCWM area, number of spinal cord T2 lesions, age, sex and disease duration^2^. Another study showed SCGM atrophy to be a biomarker for predicting disability in amyotrophic lateral sclerosis^3^.

The ability to automatically assess and characterize these changes is, therefore, an important step^4^ in the modern pipeline to study both the *in vivo* and *ex vivo* SC. The segmentation outcome can also be used for co-registration and spatial normalization to a common space. Moreover, the fully-automated segmentation is useful for longitudinal studies, where the delineation of gray matter is time consuming^4^.

While recent cervical cord cross-sectional area (CSA) segmentation methods have achieved near-human performance^5^, the accurate segmentation of the GM remains a challenge^6^. The main properties that make the GM area difficult to segment are: inconsistent intensities of the surrounding tissues, image artifacts and pathology-induced changes in the image contrast^4^.

**Figure 1:**
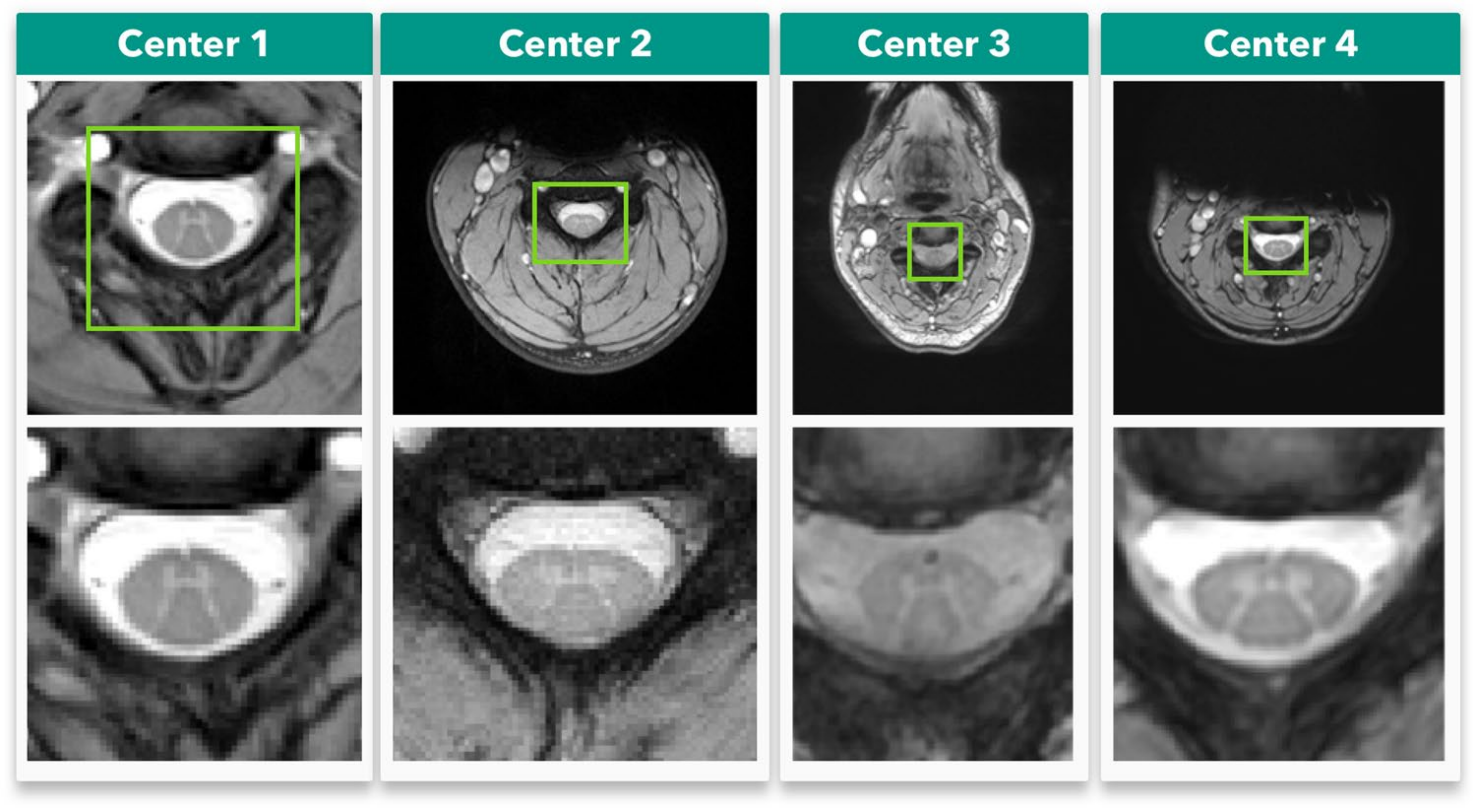
*In vivo* axial-slice samples from four centers (UCL, Montreal, Zurich, Vanderbilt) that collaborated to the SCGM Segmentation Challenge^6^. Top row: original MRI images. Bottom row: a crop of the spinal cord (green rectangle).

Additional factors also contribute to the complexity of the GM segmentation task, such as lack of standardized datasets, differences in MRI acquisition protocols, different pixel sizes, different methods to acquire gold standard segmentations and different performance metrics to assess segmentation results^6^. Figure 1 features several examples of axial MRI acquired at different centers, demonstrating image variability due variable image acquisition systems and protocols.

Despite these difficulties, there have been major improvements in acquisition and analysis methods in recent years, making it possible to obtain reliable GM segmentations. From the acquisition standpoint, the advances in coil sensitivity^7^, multi-echo gradient echo sequences^8^, and phase-sensitive inversion recovery sequences^9^ drastically improved the contrast-to-noise-ratio between the white and gray matter in the cord. From the analysis standpoint, the scientific community recently organized a collaboration effort called “Spinal Cord Gray Matter Segmentation Challenge” (SCGM Challenge)^6^ to characterize the state-of-the-art and compare six independent developed methods^10–15^ on a public available standard dataset created through the collaboration of four internationally recognized spinal cord imaging research groups (University College London, Polytechnique Montreal, University of Zurich and Vanderbilt University), providing therefore a ground basis for method comparison that was previously unfeasible.

In the past few years, we have witnessed the fast and unprecedented development of Deep Learning^16^ methods, that have not only achieved human-level performance but, in many cases, have surpassed it^17^, even in health domain applications^18^. After the results presented in the seminal paper of the AlexNet^19^, the Machine Learning community embraced the successful Deep Learning approach for Machine Learning and, consequently, many methods have been developed to since become the state-of-the-art and pervasive in many different fields such as image classification^20^, image segmentation^21^, speech recognition^22^, natural language processing (NLP), among others.

Deep Learning is characterized by a major shift from traditional handcraft feature extraction to a hierarchical representation learning approach where multiple levels of automatically discovered representations are learned from raw data^16^.

In a recent survey^23^ of over 300 papers that used Deep Learning techniques for medical image analysis, the authors found that these techniques have spread throughout the entire field of medical image analysis, with a rapid increase in the number of publications between the years of 2015 and 2016. The survey also found that Convolutional Neural Networks (CNNs) were more prevalent in the medical image analysis, with Recurrent Neural Networks (RNNs) gaining more popularity.

Although the enormous success of Deep Learning has attracted a lot of attention from the research community, some challenges in the medical imaging domain remain open, such as data acquisition, which is usually very expensive and requires time-consuming annotation from image specialists to create the gold standards necessary for algorithm training and validation. Standardized datasets remain also a major problem due to variability in equipment from different vendors, acquisition protocols/parameters/contrasts, especially in the MRI domain. Furthermore, data availability is limited due to concerns around ethics and regulations on patient data privacy^23^.

In this work, we propose a new simple pipeline featuring an end-to-end learning approach for fully-automated spinal cord gray matter segmentation using a novel Deep Learning architecture based on the *Atrous* Spatial Pyramid Pooling (ASPP)^21,24^, where we achieved state-of-the-art results on many metrics in an *in vivo* independent dataset evaluation. We further demonstrate an excellent generalization on an *ex vivo* high-resolution acquisition dataset where only a few axial-slices were annotated to accurate segment an MRI volume with more than 4000 axial slices. Our proposed method is compared with the commonly used U-Net^25^ architecture and with six other independently developed methods.

This work was implemented as the *sct_deepseg_gm* tool in the Spinal Cord Toolbox (SCT)^26^ and is now freely available at SCT Github repository in https://github.com/neuropoly/spinalcordtoolbox. SCT is a comprehensive, free and open-source library of analysis tools for MRI of the spinal cord.

## Related Work

Many methods for spinal cord segmentation were proposed in the past. Regarding the presence or absence of manual intervention, the segmentation methods can be separated into two main categories: semi-automated and fully-automated.

In the work^14^, they propose a probabilistic method for segmentation called “Semi-supervised VBEM”, whereby the observed MRI signals are assumed to be generated by the warping of an averagely shaped reference anatomy^6^. The observed image intensities are modeled as random variables drawn from a Gaussian mixture distribution, where the parameters are estimated using a variational version of the Expectation-Maximization (EM)^14^ algorithm. The method can be used in a fully unsupervised fashion or by incorporating training data with manual labels, hence the semi-supervised scheme^6^.

The SCT (Spinal Cord Toolbox) segmentation method^13^, uses an atlas-based approach and was built based on a previous work^27^ but with additional improvements such as the use of vertebral level information and linear intensity normalization to accommodate multi-site data^13^. The SCT approach first builds a dictionary of images using manual WM/GM segmentations after a pre-processing step, then the target image is also pre-processed and normalized, after that, the target image is projected into the PCA (Principal Component Analysis) space of the dictionary images where the most similar dictionary slices are selected using an arbitrary threshold. Finally, the segmentation is done using label fusion between the manual segmentations from the dictionary images that were selected^6^. The SCT method is freely available as open-source software at https://github.com/neuropoly/spinalcordtoolbox ^26^.

In the work^10^, a method called “Joint collaboration for spinal cord gray matter segmentation” (JCSCS) is proposed, where two existing label fusion segmentation methods were combined. The method is based on a multi-atlas segmentation propagation using registration and segmentation in 2D slice-wise space. In JCSCS, the “Optimized PatchMatch Label Fusion” (OPAL)^28^ is used to detect the spinal cord, where the cord localization is achieved by providing an external dataset of spinal cord volumes and their associated manual segmentation^10^, after that, the “Similarity and Truth Estimation for Propagated Segmentations” (STEPS)^29^ is used to segment the GM in two steps, first the segmentation propagation, and then a consensus segmentation is created by fusing best-deformed templates (based on locally normalized cross-correlation)^10^.

In the work^12^, the Morphological Geodesic Active Contour (MGAC) algorithm uses an external spinal cord segmentation tool (“Jim”, from Xinapse Systems) to estimate the spinal cord boundary and a morphological geodesic active contour model to segment the gray matter. The method has five steps: first, the original image spinal cord is segmented with the Jim software and then a template is registered to the subject cord, after which the same transformation is applied to the GM template. The transformed gray matter template is then used as an initial guess for the active contour algorithm^12^.

The “Gray matter Segmentation Based on Maximum Entropy” (GSBME) algorithm^6^ is a semi-automatic, supervised segmentation method for the GM. The GSBME is comprised of three main stages. First, the image is pre-processed, in this step the GSBME uses the SCT^26^ to segment the spinal cord using Propseg^5^ with manual initialization, after which the image intensities are normalized and denoised. In the second step, the images are thresholded, slice by slice, using a sliding window where the optimal threshold is found by maximizing the sum of the GM and WM intensity entropies. In the final stage, an outlier detector discards segmented intensities using morphological features such as perimeter, eccentricity and Hu moments among others^6^.

In the Deepseg approach^15^, which builds upon the work^11^, a Deep Learning architecture similar to the U-Net^25^, where a CNN has a contracting and expanding path. The contracting path aggregates information while the expanding path upsamples the feature maps in order to achieve a dense prediction output. To recover spatial information loss, shortcuts are added between contracting/expanding paths of the network. In Deepseg, instead of using upsampling layers like U-Net, they use an unpooling and “deconvolution” approach such as in the work^30^. The network architecture possesses 11 layers and is pre-trained using 3 convolutional restricted Boltzmann Machines^31^. Deepseg also uses a loss function with a weighted sum of two different terms, the mean square differences of the GM and non-GM voxels, thus balancing sensitivity and specificity^6^. Two models were trained independently, one for the full spinal cord segmentation and another for the GM segmentation.

We compare our method with all the aforementioned methods on the SCGM Challenge^6^ dataset.

## Methods and Materials

As in the *Related Work* section, the majority of the previously developed GM segmentation methods usually rely on registered templates/atlases, arbitrary distance and similarity metrics, and/or complex pipelines that are not optimized in an end-to-end fashion and neither efficient during inference time.

In this work, we focus on the development of a simple Deep Learning method that can be trained in an end-to-end fashion and that generalizes well even with a small subset of 2D labeled axial slices belonging to a larger 3D MRI volume.

### Note on U-Nets

Many modern Deep Learning CNN classification architectures use alternating layers of convolutions and subsampling operations to aggregate semantic information and discard spatial information across the network, leading to certain levels of translation and rotation invariance that are important for classification. However, in segmentation tasks, a dense full-resolution output is required. In medical imaging, the most established architecture for segmentation is the well-known U-Net^25^, where two distinct paths (encoder-decoder/contracting-expanding) are used to aggregate semantic information and recover the spatial information with the help of shortcut connections between the paths.

The U-Net architecture, however, causes a major expansion of the parameter space due to the two distinct paths that form the U-shape. As noted previously^32^, the gradient flow in the high-level layers of the U-Nets (bottom of the U-shape) is problematic. Since the final low-level layers have access to the earlier low-level features, the network optimization will find the shortest path to minimize the loss, thus reducing the gradient flow in the bottom of the network.

By visualizing feature maps from the U-Net using techniques described in the work^33^, we found that the features extracted in the bottom of the network were very noisy, while the features extracted in the low-level layers were the only ones that exhibited meaningful patterns. By removing the bottom layers of the network, we found that the network performed the same as, or occasionally better than, the deeper network.

### Proposed method

Our method is based on the state-of-the-art segmentation architecture called “*Atrous* Spatial Pyramid Pooling” (ASPP)^21^ that uses “*Atrous* convolutions”, also called “dilated convolutions”^34^. We performed modifications to improve the segmentation performance on medical imaging by handling imbalanced data with a different loss function, and also by extensively removing decimation operations from the network such as pooling, trading depth (due to memory constraints) to improve the equivariance of the network and also parameter reduction.

**Figure 2:**
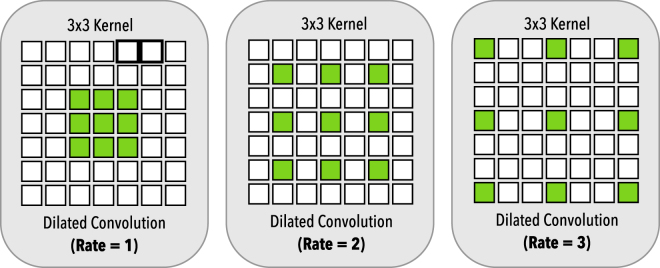
Dilated convolution. On the left, we have the dilated convolution with dilation rate *r* = 1, equivalent to the standard convolution. In the middle with have a dilation *r* = 2 and in the right a dilation rate of *r* = 3. All dilated convolutions have a 3 × 3 kernel size and the same number of parameters.

Dilated convolutions allow us to exponentially grow the receptive field with a linearly increasing number of parameters, providing a significant parameter reduction while increasing the effective receptive field^35^ and preserving the input resolution throughout the network, in contrast to wide stride convolutions where the resolution is lost. Dilated convolutions work by introducing “holes”^24^ in the kernel as illustrated in Fig. 2. For 1D signal *x*[*i*], the *y*[*i*] output of a dilated convolution with the dilation rate *r* and a filter *w*[*s*] with size *S* is formulated as:

1 $$y[i]=\sum _{s=1}^{S}x[i+r\cdot s]w[s\mathrm{]}.$$

The dilation rate *r* can also be seen as the stride over which the input signal is sampled^24^. Dilated convolutions, like standard convolutions, also have the advantage of being translationally equivalent, which means that translating the image will result in a translated version of the original input, as seen below:

2 $$f(g(x))=g(f(x))$$

where $g(\,\cdot \,)$ is a translation operation and $f(\,\cdot \,)$ a convolution operation. However, since we don’t need to introduce pooling to capture multi-scale features when using dilated convolutions, we can keep the translational equivariance property in the network, which is important for spatially dense prediction tasks, given that a translation of the input features should result also in an equivalent translation of outputs.

**Figure 3:**
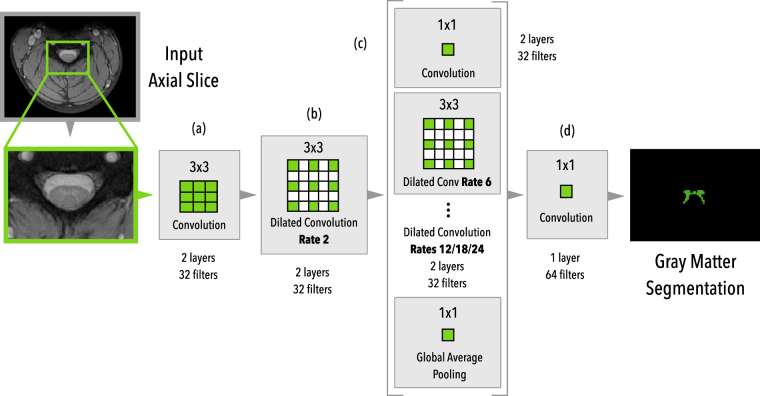
Architecture overview of the proposed method. The MRI axial slice is fed to the first block of 3 × 3 convolutions and then to a block of dilated convolutions (rate 2). Then, six parallel modules with different rates (6/12/18/24), 1 × 1 convolution, and a global average pooling are used in parallel. After the parallel modules, all feature maps are concatenated and then fed into the final block of 1 × 1 convolutions to produce the final dense predictions.

The overall proposed architecture can be seen in Fig. 3. Our architecture works with 2D slice-wise axial images and is composed of (a) two initial layers of standard 3 × 3 convolutions, followed by (b) two layers of dilated convolutions with rate *r* = 2, followed by (c) six parallel branches with two layers each of a 1 × 1 standard convolution, 4 different dilated convolution rates (6/12/18/24) and a global averaging pooling that is repeated at every spatial position of the feature map. After that, the feature maps from the six parallel branches are concatenated and forwarded to (d) a block of 2 layers with 1 × 1 convolutions in order to produce the final dense prediction probability map. Each layer is followed by Batch Normalization^36^ and Dropout^37^ layers and we did not employ residual connections.

**Figure 4:**
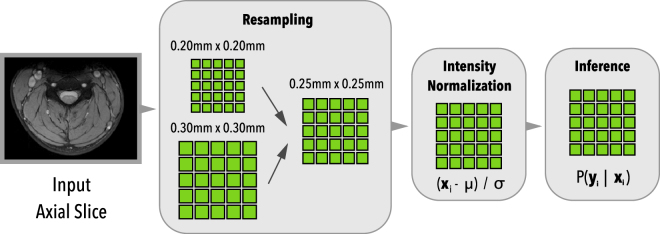
Architecture pipeline overview. During the first stage, input axial slices are resampled to a common pixel size space, then intensity is normalized, followed by the network inference.

Figure 4 illustrates the pipeline of our training/inference process. An initial resampling step downsamples/upsamples the input axial slice images to a common pixel size space, then a simple intensity normalization is applied to the image, followed by the network inference stage.

Contrary to the task of natural images segmentation, the task of GM segmentation in medical imaging is usually very unbalanced. In our case, only a small portion of the entire axial slice encompasses the GM (the rest being comprised of other structures such as the white matter, cerebrospinal fluid, bones, muscles, etc.). Due to this imbalance, we employed a surrogate loss for the DSC (Dice Similarity Coefficient) called the Dice Loss, which is insensitive to imbalancing and was employed by many works in medical imaging^38,39^. The Dice Loss can be formulated as:

3 $${ {\mathcal L} }_{dice}=\,-\,\frac{2{\sum }_{n\mathrm{=1}}^{N}{p}_{n}{r}_{n}+\varepsilon }{{\sum }_{n\mathrm{=1}}^{N}{p}_{n}+{\sum }_{n\mathrm{=1}}^{N}{r}_{n}+\varepsilon }$$

where *p* and *r* are the predictions and gold standard, respectively. The *ε* term is used to ensure loss stability by avoiding numerical issues. We experimentally found that the Dice Loss yielded better results when compared to the weighted cross-entropy (WCE) used by the original U-Net^25^, which is more difficult to optimize due to the added weighting hyper-parameter.

Medical image datasets are usually smaller than natural image datasets by many orders of magnitude, therefore regularization and data augmentation is an important step. In this work, the following data augmentation strategies were applied: rotation, shifting, scaling, flipping, noise, and elastic deformation^40^.

The main differences when we compare our proposed architecture with that of the work^21^, are the following:

**Initial pooling/decimation:** Our network does not use initial pooling layers as we found them detrimental to the segmentation of medical images;

**Padding:** We extensively employ padding across the entire network to keep feature map sizes fixed, trading depth to reduce memory usage of the network;

**Dilation Rates:** Since we do not use initial pooling, we retain the parallel dilated convolution branch with the rate *r* = 24. As we found improvements by doing so, due to the large feature map size that doesn’t cause filter degeneration as seen in^21^;

**Loss:** Contrary to natural images, our task of GM segmentation is highly unbalanced, therefore instead of using the traditional cross-entropy, we use the Dice Loss;

**Data Augmentation:** In this work we apply rotation, shifting, added channel noise, and elastic deformations^40^, in addition to the scaling and flipping used previously^21^.

Table 1 compares the setup parameters of our approach as well as the participant methods of the SCGM Segmentation Challenge^6^.

**Table 1:** Parameters of each compared method.

Method	Init.	Training	External data	Time p/slice
JCSCS	Auto.	No	Yes	4–5 min
DEEPSEG	Auto.	Yes (4 h)	No	<1 s
MGAC	Auto.	No	No	1 s
GSBME	Manual	Yes (<1 m)	No	5–80 s
SCT	Auto.	No	Yes	8–10 s
VBEM	Auto.	No	No	5 s
Proposed	Auto.	Yes (19 h)	No	<1 s

Time per slice is an estimated value, since different hardware were employed by the different techniques. Values replicated from the work^6^.

#### U-Net architecture

For the U-Net^25^ architecture model that was used for comparison, we employed a 14-layers network using standard 3 × 3 2D convolution filters with ReLU non-linearity activations^17^. For a fair comparison, we used the same training protocol and loss function. For the data augmentation strategy, we employed a more aggressive augmentation due to overfitting issues with the U-Net (see the Discussion section). We also performed an extensive architecture exploration and used the best performing U-Net model architecture.

### Datasets

In this subsection, we present the datasets used for evaluation in this work.

#### Spinal Cord Gray Matter Challenge

The Spinal Cord Gray Matter Challenge^6^ (SCGM Challenge) dataset consists of 80 healthy subjects (20 subjects from each center). The demographics range from a mean age of 28.3 up to 44.3 years old. Three different MRI systems were used (Philips Achieva, Siemens Trio, Siemens Skyra) with different acquisition parameters based on a multi-echo gradient echo sequence. The voxel size range from 0.25 × 0.25 × 2.5 mm up to 0.5 × 0.5 × 5.0 mm. The dataset is split between training (40) and test (40) with the test set hidden. For each labeled slice in the dataset, 4 gold standard segmentation masks were produced by 4 independent expert raters (one per site). Examples of the datasets from each center are shown in Fig. 1.

During the development of this work, we found some misclassified voxels in the training set. These issues were reported, however, for the sake of a fair comparison, all the evaluations done in this work used the original pristine training dataset.

#### *Ex vivo* high-resolution spinal cord

To evaluate our method on an *ex vivo* dataset, we used an MRI acquisition that was performed on an entire human spinal cord^41^, from the pyramidal decussation to the *cauda equina* using a 7 T horizontal-bore small animal MRI system.

MR images of the entire spinal cord were acquired in seven separate overlapping segments. The segment field of view was 8 × 2 × 2 cm with 1 cm of overlap on each end. Between each acquisition, the specimen was advanced precisely 7 cm through the magnet bore using a custom-machined gantry insert. T2*-weighted anatomic images were acquired using a 3D gradient echo sequence with an acquisition matrix of 1600 × 400 × 400, resulting in 50 micron isotropic resolution. Scan parameters included: TR = 50 ms, TE = 9 ms, flip angle = 60°, bandwidth = 100 kHz and number of averages = 1. Per-segment acquisition time was 2 hours 22 minutes, resulting in a total acquisition time of approximately 16 hours. Individual image segments were composed into a single volume using automated image registration and weighted averaging of overlapping segments.

Although the acquisition was obtained from a deceased adult male with no known history of neurologic disease, the review of images revealed a clinically occult SC lesion close to the 6th thoracic nerve root level, with imaging features suggestive of a chronic compressive myelopathy or possible sequela of a previous viral infection such as herpes zoster.

The volume is comprised of a total 4676 axial slices with 100 isotropic resolution.

The annotations (gold standard) for axial slices of this dataset were made by a researcher with the help of an expert radiologist. The annotation procedure was as follows: first, the contour of the GM was delineated using a gradient method from MIPAV^42^ software. After that, a pixel-wise fine-tuning was performed using the fslview tool from FSL^43^.

### Training Protocol

#### Spinal Cord Gray Matter Challenge

The training protocol for the SCGM Challenge^6^ dataset experiments are described in Table 2 and the data augmentation parameters are described in Table 3.

**Table 2:** Training protocol for the Spinal Cord Gray Matter Challenge dataset.

Resampling and Cropping	All volumes were resampled to a voxel size of 0.25 × 0.25 mm, the highest resolution found between acquisitions. All the axial slices were center-cropped to a 200 × 200 pixels size.
Normalization	We performed only mean centering and standard deviation normalization of the volume intensities.
Train/validation split	For the train/validation split, we used 8 subjects (2 from each site) for validation and the rest for training. The test set was defined by the challenge. We haven’t employed any external data or used the vertebral information from the provided dataset. Only the provided GM masks were used for training/validation.
Batch size	We used a small batch size of only 11 samples.
Optimization	We used Adam^48^ optimizer with a small learning rate $\eta =0.001$.
Batch Normalization	We used a momentum $\varphi =0.1$ for BatchNorm due to the small batch size.
Dropout	We used a dropout rate of 0.4.
Learning Rate Scheduling	Similar to the work^21^, we used the “poly” learning rate policy where the learning rate is defined by $\eta ={\eta }_{{t}_{0}}\ast {(1-\frac{n}{N})}^{p}$ where ${\eta }_{{t}_{0}}$ is the initial learning rate, *N* is the number of epochs, *n* the current epoch and *p* the power with *p* = 0.9.
Iterations	We trained the model for 1000 epochs (w/ 32 batches at each epoch).
Data augmentation	We applied the following data augmentations: rotation, shift, scaling, channel shift, flipping and elastic deformation^40^. The data augmentation parameters were chosen using random search. More details about the parameters of the data augmentation are presented in Table 3.

**Table 3:** Data augmentation parameters used during the training stage of the Spinal Cord Gray Matter Challenge dataset.

Augmentation	Parameter	Probability
Rotation (degrees)	[−4.6, 4.6]	0.5
Shift (%)	[−0.03, 0.03]	0.5
Scaling	[0.98, 1.02]	0.5
Channel Shift	[−0.17, +0.17]	0.5
Elastic Deformation^40^	$\alpha =30.0$, $\sigma =4.0$	0.3

Contrary to the smooth decision boundaries characteristic of models trained using cross-entropy, the Dice Loss has the property of creating sharp decision boundaries and models with high recall rate. We found experimentally that thresholding the dense predictions with a threshold *τ* = 0.999 provided a good compromise between precision/recall, however, no optimization was employed to choose the threshold *τ* value for the output predictions.

Since the test dataset is hidden from the challenge participants, to evaluate our model we sent our produced test predictions to the challenge website for automated evaluation. Results are presented in Table 4 under the column “Proposed Method”, alongside with the six other previously developed methods and 10 different metrics.

**Table 4:** Comparison of different segmentation methods that participated in the SCGM Segmentation Challenge^6^ against each of the four manual segmentation masks of the test set, reported here in the format: mean (std).

	JCSCS	DEEPSEG	MGAC	GSBME	SCT	VBEM	Proposed Method
DSC	0.79 (0.04)	0.80 (0.06)	0.75 (0.07)	0.76 (0.06)	0.69 (0.07)	0.61 (0.13)	**0.85** (0.04)
MSD	0.39 (0.44)	0.46 (0.48)	0.70 (0.79)	0.62 (0.64)	0.69 (0.76)	1.04 (1.14)	**0.36** (0.34)
HSD	2.65 (3.40)	4.07 (3.27)	3.56 (1.34)	4.92 (3.30)	3.26 (1.35)	5.34 (15.35)	**2.61** (2.15)
SHD	1.00 (0.35)	1.26 (0.65)	1.07 (0.37)	1.86 (0.85)	1.12 (0.41)	2.77 (8.10)	**0.85** (0.32)
SMD	0.37 (0.18)	0.45 (0.20)	0.39 (0.17)	0.61 (0.35)	0.39 (0.16)	0.54 (0.25)	**0.36** (0.17)
TPR	77.98 (4.88)	78.89 (10.33)	87.51 (6.65)	75.69 (8.08)	70.29 (6.76)	65.66 (14.39)	**94.97** (3.50)
TNR	**99.98** (0.03)	99.97 (0.04)	99.94 (0.08)	99.97 (0.05)	99.95 (0.06)	99.93 (0.09)	99.95 (0.06)
PPV	81.06 (5.97)	**82.78** (5.19)	65.60 (9.01)	76.26 (7.41)	67.87 (8.62)	59.07 (13.69)	77.29 (6.46)
JI	0.66 (0.05)	0.68 (0.08)	0.60 (0.08)	0.61 (0.08)	0.53 (0.08)	0.45 (0.13)	**0.74** (0.06)
CC	47.17 (11.87)	49.52 (20.29)	29.36 (29.53)	33.69 (24.23)	6.46 (30.59)	−44.25 (90.61)	**64.24** (10.83)

For of fair comparison, the metrics are the same as used in the study^6^ and the results from other methods are replicated here, where we have: Dice similarity coefficient (DSC), mean surface distance (MSD), Hausdorff surface distance (HSD), skeletonized Hausdorff distance (SHD), skeletonized median distance (SMD), true positive rate (TPR), true negative rate (TNR), positive predictive value (PPV), Jaccard index (JI) and conformity coefficient (CC). In bold font, we represent the best-obtained results on each metric. We also note that MSD, HSD, SHD and SMD metrics are in millimeters and that lower values mean better results.

The training time on a single NVIDIA P100 GPU took approximately 19 hours using single-precision floating-point and TensorFlow 1.3.0 with cuDNN 6, while inference time took less than 1 second per subject.

#### Inter-rater variability as label smoothing regularization

The training dataset provided by the SCGM Challenge is comprised of 4 different masks that were manually and independently created by raters for each axial slice. As in the study^11^, we used all the different masks as our gold standard. We also found that this approach shares the same principle of using label smoothing as seen in work^44^.

Label smoothing is a mechanism that has the effect of reducing the confidence of the model by preventing the network from assigning a full probability to a single class, which is commonly evidence of overfitting. In the study^45^, a link was found between label smoothing and the confidence penalty through the direction of the Kullback-Leibler divergence. Since the different gold standard masks for the same axial slices diverges usually only in the border of the GM, it is easy to see that this has a label smoothing effect on the contour of the GM, thereby encouraging the model to be less confident in the contour prediction of the GM, a kind of “empirical contour smoothing”.

This interpretation suggests that one could also incorporate this contour smoothing by artificially adding label smoothing on the contours of the target anatomy, where raters usually disagree on the manual segmentation, leading to a potentially better model generalization on many different medical segmentation tasks where the contours are usually the region of raters disagreement.

We leave the exploration of this contour smoothing to future work.

#### *Ex vivo* high-resolution spinal cord

The training protocol for the *ex vivo* high-resolution spinal cord dataset experiments are described in Table 5 and the data augmentation parameters are described in the Table 6.

**Table 5:** Training protocol for the *Ex vivo* high-resolution spinal cord dataset.

Cropping	All the slices were center-cropped to a 200 × 200 pixels size.
Normalization	We performed only mean centering and standard deviation normalization of the volume intensities.
Train/validation split	For the training set we selected only 15 evenly spaced axial slices out of 4676 total slices from the volume. For the validation set, we selected 7 (evenly spaced) axial slices and our test set was comprised of 8 axial slices (also evenly distributed across the entire volume).
Batch size	We used a small batch size of only 11 samples.
Optimization	We used Adam^48^ optimizer with a small learning rate $\eta =0.001$.
Batch Normalization	We used a momentum $\varphi =0.1$ for BatchNorm due to the small batch size.
Dropout	We used a dropout rate of 0.4.
Learning Rate Scheduling	Similar to the work^21^, we used the “poly” learning rate policy where the learning rate is defined by $\eta ={\eta }_{{t}_{0}}\ast {(1-\frac{n}{N})}^{p}$ where ${\eta }_{{t}_{0}}$ is the initial learning rate, *N* is the number of epochs, *n* the current epoch and *p* the power with $p=0.9$.
Iterations	We trained the model for 600 epochs (w/ 32 batches at each epoch).
Data augmentation	For this dataset, we used the following aforementioned augmentations: rotation, shift, scaling, channel shift, flipping and elastic deformation^40^. We didn’t employed random search to avoid overfitting due to the dataset size. More details about the parameters of the data augmentation are presented in Table 6.

**Table 6:** Data augmentation parameters used during the training stage of the *Ex vivo* high-resolution spinal cord dataset.

Augmentation	Parameter	Probability
Rotation (degrees)	[−5.0, 5.0]	0.5
Shift (%)	[−0.1, 0.1]	0.5
Scaling	[0.9, 1.1]	0.5
Channel Shift	[−0.3, +0.3]	0.5
Flipping	Horizontal	0.5
Elastic Deformation^40^	$\alpha =30.0$, $\sigma =4.0$	0.3

Like in the SCGM Segmentation task, we used a threshold *τ* = 0.999 to binarize the prediction mask.

The training time on a single NVIDIA P100 GPU took approximately 2 hours using single-precision floating-point and TensorFlow 1.3.0 with cuDNN 6. While inference time took approximately 25 seconds to segment 4676 axial slices.

### Data availability

The SCGM Challenge dataset analyzed during the current study is available on the SCGM Challenge repository at http://rebrand.ly/scgmchallenge. The *ex vivo* dataset analyzed during the current study is not publicly available, but is available from the corresponding author on reasonable request.

## Results

In this section, we discuss the experimental evaluation of the method in the presented datasets.

### Spinal Cord Gray Matter Challenge

In this subsection, we show the evaluation on SCGM Challenge^6^ dataset.

#### Qualitative Evaluation

**Figure 5:**
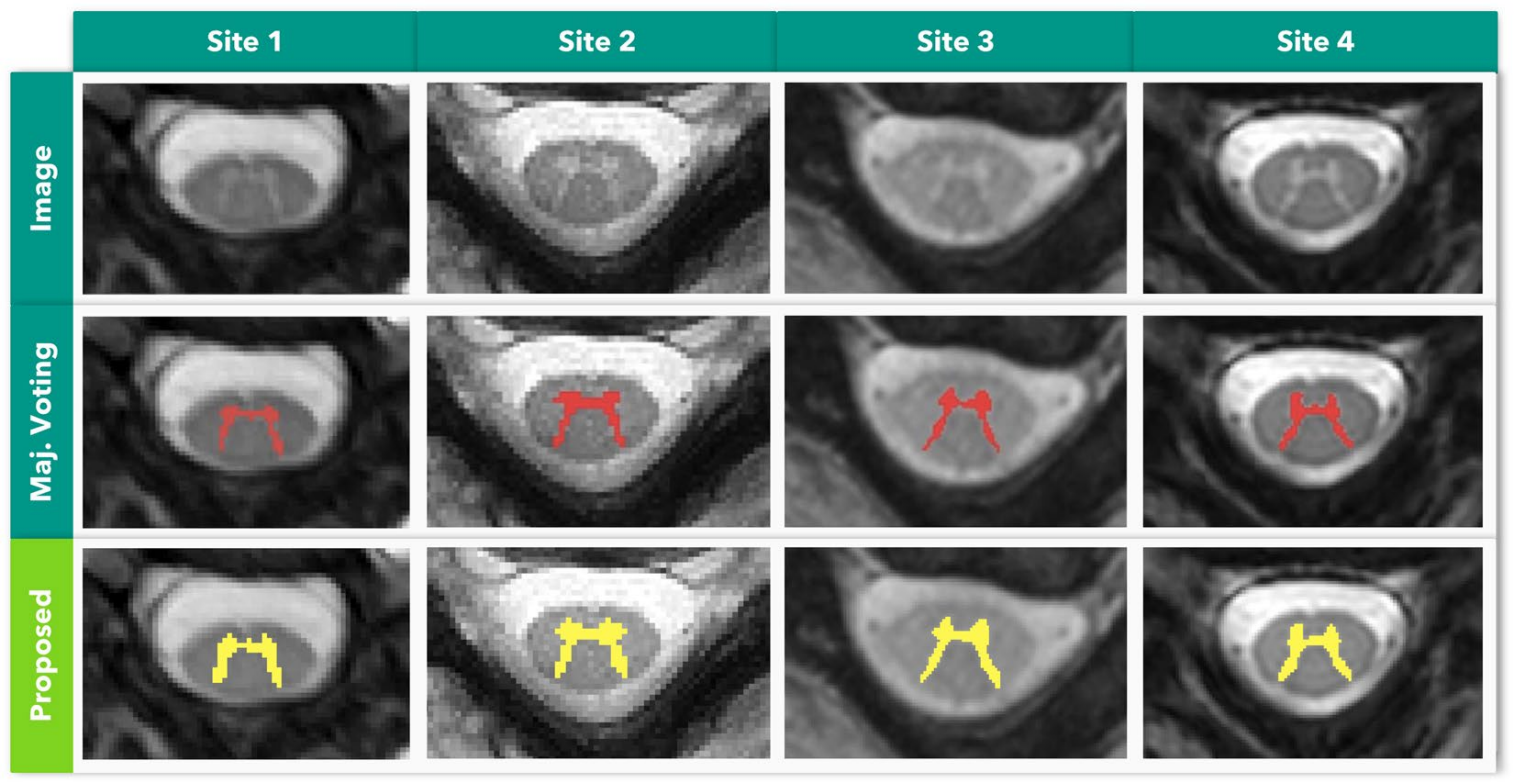
Qualitative evaluation of our proposed approach on the same axial slice for subject 11 of each site. From top to bottom row: input image, majority voting segmentation gold standard, and the result of our segmentation method. Adapted from the work^6^.

In Fig. 5, we show the segmentation output of our model in four different subjects, from acquisitions performed at the four different centers, on the test set of the SCGM Segmentation Challenge. The majority voting segmentation was taken from the study^6^. As we can see in Fig. 5, our approach was able to capture many properties of the GM anatomy, providing good segmentations even in presence of blur, as seen in the samples from Site 1 and Site 3.

When compared with the segmentation results from Deepseg^15^, that uses a U-Net like structure with pre-training and 3D-wise training, we can see that our method succeeds at segmenting the gray commissure of the GM structure, which was observed to pose a problem for Deepseg, as indicated in Fig. 4 of the work^6^.

#### Quantitative Evaluation

**Figure 6:**
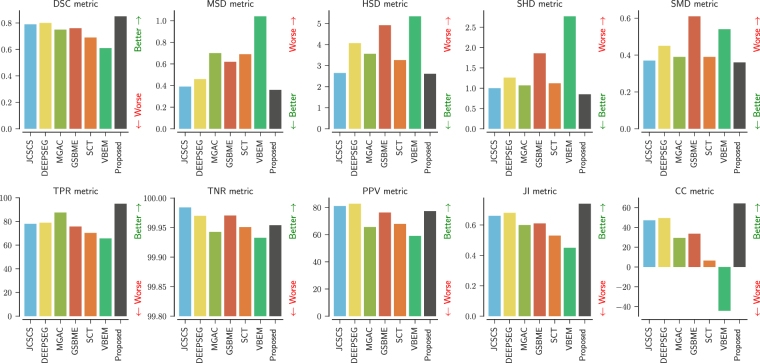
Test set evaluation results from the SCGM segmentation challenge^6^ for each evaluated metric, with the Dice similarity coefficient (DSC), mean surface distance (MSD), Hausdorff surface distance (HSD), skeletonized Hausdorff distance (SHD), skeletonized median distance (SMD), true positive rate (TPR), true negative rate (TNR), positive predictive value (PPV), Jaccard index (JI) and conformity coefficient (CC). Our method is shown as “Proposed”. Best viewed in color.

As we can see in Table 4 and Fig. 6, our approach achieved state-of-the-art results in 8 out of 10 different metrics and surpassed 4 out of 6 previous methods on all metrics. A description of the metrics used in this work is given in Table 7.

**Table 7:** Description of the validation metrics. Adapted from the work^6^.

Metric Name	Abbr.	Range	Interpretation	Category
Dice Similarity Coefficient	DSC	0–1	Similarity between masks	Overlap
Jaccard Index	JI	0–100	Similarity between masks	Overlap
Conformity Coefficient	CC	<100	Ratio between mis-segmented and correctly segmented	Overlap
Symmetric Mean Absolute Surface Distance	MSD	>0	Mean euclidean distance between mask contours (mean error)	Distance
Hausdorff Surface Distance	HSD	>0	Longest euclidean distance between mask contours (absolute error)	Distance
Skeletonized Hausdorff Distance	SHD	>0	Indicator of maximal local error	Distance
Skeletonized Median Distance	SMD	>0	Indicator of global errors	Distance
True Positive Rate or Sensitivity	TPR	0–100	Low values mean that method tends to under-segment	Statistical
True Negative Rate or Specificity	TNR	0–100	Quality of segmented background	Statistical
Positive Predictive Value or Precision	PPV	0–100	Low values mean that method tends to over-segment	Statistical

We can also see that the Dice Loss is not only an excellent surrogate for the Dice Similarity Coefficient (DSC) but also a surrogate for distance metrics, as we note that our model not only achieved state-of-the-art results on overlap metrics (i.e. DSC) but also on distance and statistical metrics.

The True Negative Rate (TNR) and Positive Predictive Value (PPV) or precision, were metrics for which the proposed method did not achieve the best results. However, we note that the TNR was very close to the results of other methods. We also hypothesize that the suboptimal results of the precision (PPV) are an effect of the sharp decision boundary produced by our model due to the Dice Loss. We are confident that the prediction threshold optimization can yield better results, however, this cost optimization would require further investigations.

When compared to the Deepseg^15^ method, the only method using Deep Learning in the challenge, where an U-Net based architecture was employed, our proposed approach performed better in 8 out of 10 metrics, even though our method did not employ 3D convolutions, pre-training, or threshold optimization as was done in Deepseg^15^.

### *Ex vivo* high-resolution spinal cord

In this subsection, we show the evaluation on the *ex vivo* high-resolution spinal cord dataset.

#### Qualitative Evaluation

**Figure 7:**
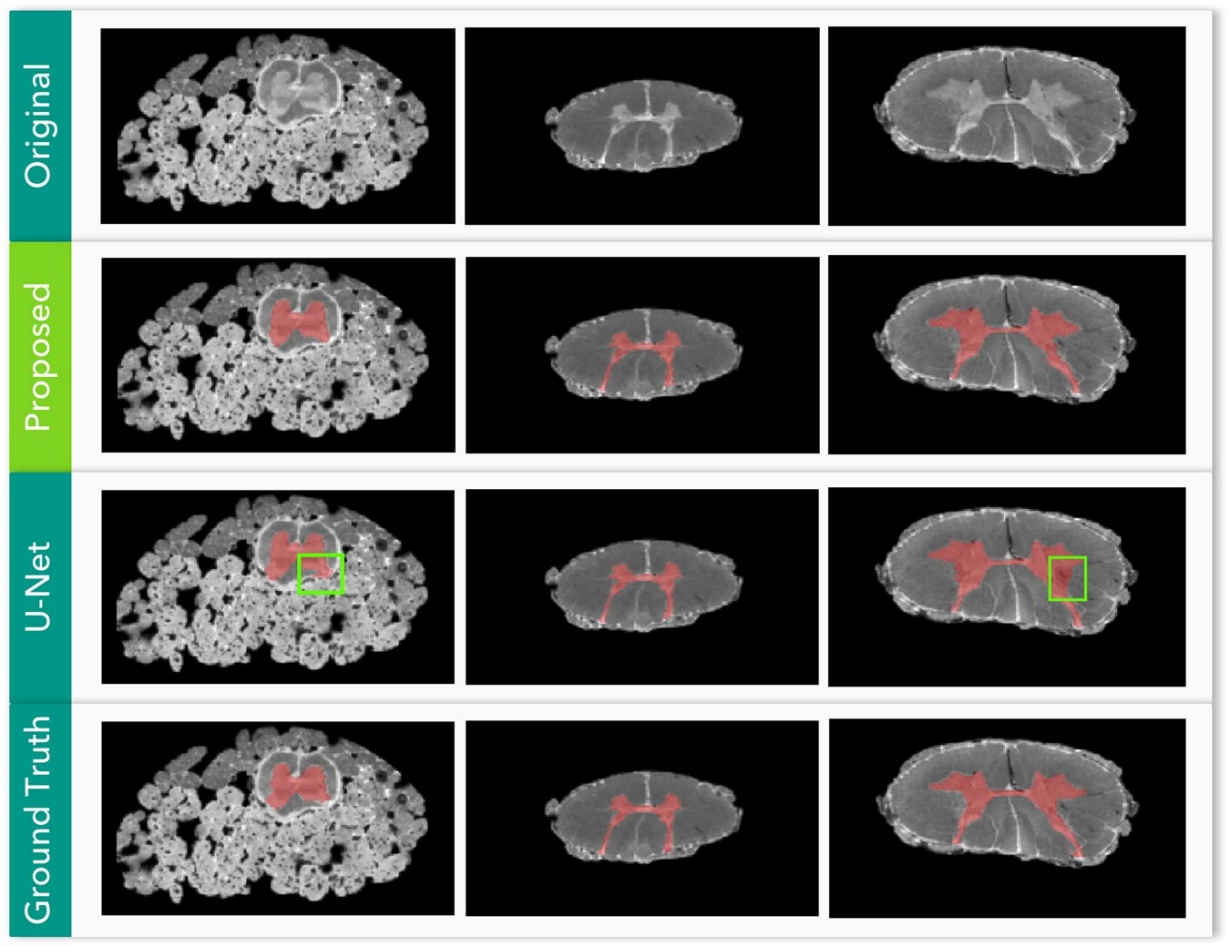
Qualitative evaluation of the U-Net and our proposed method on the *ex vivo* high-resolution spinal cord dataset. Each column represents a random sample of the test set (regions from left to right: sacral, thoracic, cervical). Green rectangles frame the oversegmentations of the U-Net model predictions.

In Fig. 7, we show a qualitative evaluation of the segmentations produced by our method and those of U-Net model, contrasting the segmentations against the original and gold standard images.

As can be seen in the test sample depicted in the first column of Fig. 7, the predictions of the U-Net “leaked” the gray matter segmentation into the cerebrospinal fluid (CSF) close to the dorsal horn (see green rectangle on first column), while our proposed segmentation was much more contained in the gray matter region only.

Also, in the third column of the Fig. 7, the U-Net significantly oversegmented a large portion of the GM region, extending the segmentation up to the white matter close to the right lateral horn of the GM anatomy (see the green rectangle), while our proposed method performed well.

**Figure 8:**
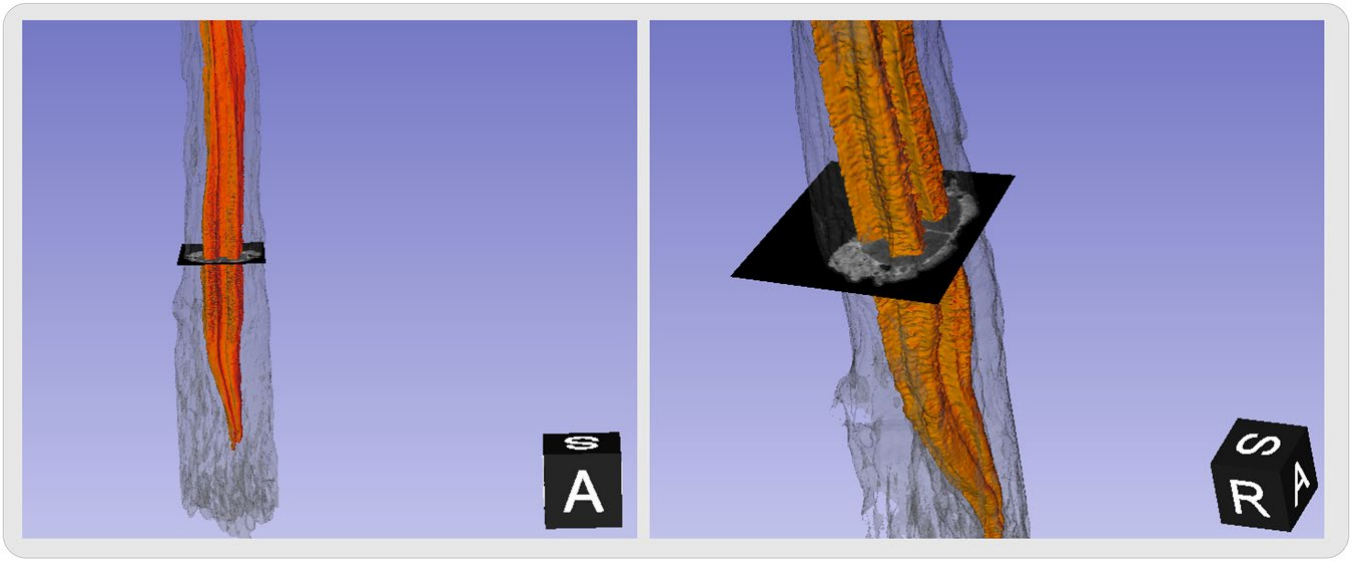
Lumbosacral region 3D rendered view of the *ex vivo* high-resolution spinal cord dataset segmented using the proposed method. The gray matter is depicted in orange color while the white matter and other tissues are represented in transparent gray color.

We also provide in Fig. 8 a 3D rendered representation of the segmented gray matter using our method.

#### Quantitative Evaluation

As seen in Table 8, which shows the quantitative results of our approach, our method achieved better results on 6 out of 8 metrics. One of the main advantages that we can see from these results is that our method uses 6 × fewer parameters than the U-Net architecture, leading to less chance of overfitting and potentially better generalization.

**Table 8:** Quantitative metric results comparing a U-Net architecture and our proposed approach on the *ex vivo* high-resolution spinal cord dataset.

Metric name	U-Net	Proposed
Num. of Params.	776,321	**124,769**
Dice	0.9027 (0.07)	**0.9226** (0.04)
Mean Accuracy	**0.9626** (0.02)	0.9561 (0.03)
Pixel Accuracy	0.9952 (0.01)	**0.9968** (0.00)
Recall	**0.9287** (0.05)	0.9135 (0.06)
Precision	0.8831 (0.10)	**0.9335** (0.04)
Freq. Weighted IU	0.9913 (0.01)	**0.9938** (0.00)
Mean IU	0.9121 (0.06)	**0.9280** (0.04)

During the training of the two architectures (U-Net and our method), we noticed that even with a high dropout rate of 0.4, the U-Net was still overfitting, forcing us to use a more aggressive data augmentation strategy to achieve better results, especially for the shifting parameters of the data augmentation; we hypothesize that this is an effect of the decimation on the contracting path of the U-Net, that disturbs the translational equivariance property of the network, leading to a poor performance on segmentation tasks.

## Discussion

In this work, we devised a simple, efficient and end-to-end method that achieves state-of-the-art results in many metrics when compared to six independently developed methods, as detailed in Table 4. To the best of our knowledge, our approach is the first to achieve better results in 8 out of the 10 metrics used in the SCGM Segmentation Challenge^6^.

One of the main differences with other methods from the challenge is that our method employs an end-to-end learning approach, whereby the entire prediction pipeline is optimized using backpropagation and gradient descent. This is in contrast to the other methods, which generally employ separate registrations, external atlases/templates data and label fusion stages. As we can also see in Table 8, when we compare our method to the most commonly used method (U-Net) for medical image segmentation, our method provides not only better results for many metrics, but also a major parameter reduction (more than 6 times).

In the lens of Minimum Description Length (MDL) theory^46^, which describes models as languages for describing properties of the data and sees inductive inference as finding regularity in the data^47^, when two competing explanations for the data explains the data well, MDL will prefer the one that provides a shorter description of the data. Our approach using dilated filters provides more than 6× parameter reduction compared to U-Nets, but is also able to outperform other methods in many metrics, an evidence that the model is parameter-efficient and that it can capture a more compact description of the data regularities when compared with more complex models such as U-Nets.

The proposed approach has been tested on data acquired using Phase-Sensitive Inversion Recovery (PSIR) sequence, and as expected, the method did not work given that the model was not trained on PSIR samples and that these data exhibit very different contrast than the T2* images the model was trained on. This can be solved by aggregating the PSIR data into the existing datasets before training the model or even by training a specific model for PSIR data. Other techniques in the field of Domain Adaptation, which is currently a very active research area, could also be useful to generalize the existing model without even requiring annotated PSIR data, depending on the technique. These investigations will be the focus of follow-up studies.

Our approach is limited to 2D slices, however, the model does not restrict the use of 3D dilated convolutions and we believe that incorporating 3D context information into the model would almost certainly improve the segmentation results, however, at the expense of increased memory consumption.

Although we believe that this method can be extended to the GM segmentation in the presence of many different neurological conditions such as multiple sclerosis, this will need to be further confirmed in follow-up studies in which patients would be included in the training/validation dataset.

We also believe that our method can be expanded to take advantage of semi-supervised learning approaches due to the strong smoothness assumption that holds for axial slices in most volumes, especially in *ex vivo* high-resolution spinal cord MRI.
